# Oxidative and Non-Oxidative Antimicrobial Activities of the Granzymes

**DOI:** 10.3389/fimmu.2021.750512

**Published:** 2021-10-11

**Authors:** Marilyne Lavergne, Maria Andrea Hernández-Castañeda, Pierre-Yves Mantel, Denis Martinvalet, Michael Walch

**Affiliations:** ^1^ Department of Oncology, Microbiology and Immunology, Anatomy Unit, Faculty of Science and Medicine, University of Fribourg, Fribourg, Switzerland; ^2^ Division Infectious Disease and International Medicine, Department of Medicine, Center for Immunology, Minneapolis, MN, United States; ^3^ Department of Biomedical Sciences, Venetian Institute of Molecular Medicine, Padova, Italy; ^4^ Department of Biomedical Sciences, University of Padua, Padova, Italy

**Keywords:** granzymes, ROS - reactive oxygen species, caspases, antimicrobial defense, apoptosis, mitochondria

## Abstract

Cell-mediated cytotoxicity is an essential immune defense mechanism to fight against viral, bacterial or parasitic infections. Upon recognition of an infected target cell, killer lymphocytes form an immunological synapse to release the content of their cytotoxic granules. Cytotoxic granules of humans contain two membrane-disrupting proteins, perforin and granulysin, as well as a homologous family of five death-inducing serine proteases, the granzymes. The granzymes, after delivery into infected host cells by the membrane disrupting proteins, may contribute to the clearance of microbial pathogens through different mechanisms. The granzymes can induce host cell apoptosis, which deprives intracellular pathogens of their protective niche, therefore limiting their replication. However, many obligate intracellular pathogens have evolved mechanisms to inhibit programed cells death. To overcome these limitations, the granzymes can exert non-cytolytic antimicrobial activities by directly degrading microbial substrates or hijacked host proteins crucial for the replication or survival of the pathogens. The granzymes may also attack factors that mediate microbial virulence, therefore directly affecting their pathogenicity. Many mechanisms applied by the granzymes to eliminate infected cells and microbial pathogens rely on the induction of reactive oxygen species. These reactive oxygen species may be directly cytotoxic or enhance death programs triggered by the granzymes. Here, in the light of the latest advances, we review the antimicrobial activities of the granzymes in regards to their cytolytic and non-cytolytic activities to inhibit pathogen replication and invasion. We also discuss how reactive oxygen species contribute to the various antimicrobial mechanisms exerted by the granzymes.

## Introduction

**Figure 1:**
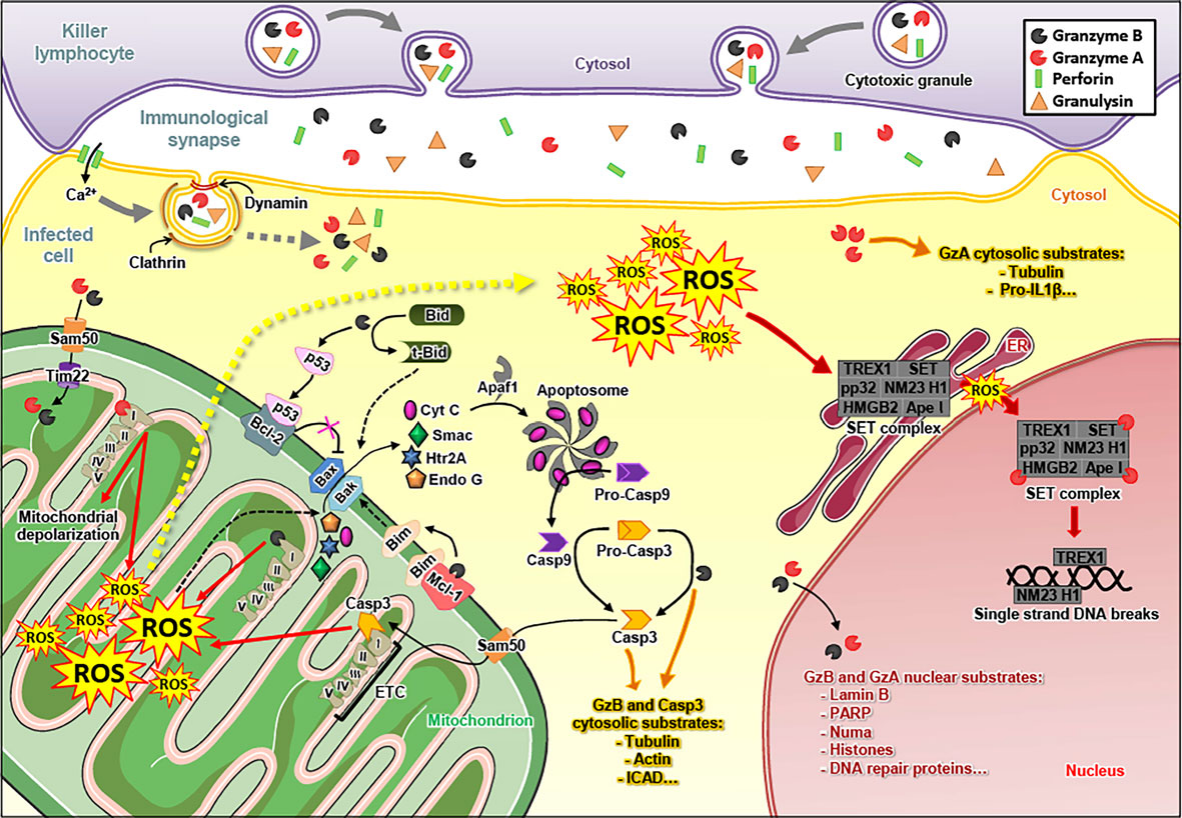
Granzyme A and Granzyme B induce apoptosis of infected cells. The killer lymphocyte releases the content of its granules in the immunological synapse, *i.e.* the immune effectors granzyme A (GzmA), granzyme B (GzmB), perforin (PFN) and granulysin (GNLY). The endocytosis of GzmA, GzmB, PFN and GNLY in the infected cell is mediated by a PFN-dependent calcium (Ca^2+^) influx and relies on clathrin and dynamin. Once in the cytosol, GzmA and GzmB cleave various substrates. GzmB triggers the formation of Bax/Bak pores in the outer mitochondrial membrane by cleaving Bid (in truncated-Bid, t-Bid), Mcl-1 (which releases Bim) (12) and p53 (which inhibits Bcl2) (13). GzmB also directly cleaves and matures pro-caspase 3 (Pro-Casp3) into active caspase 3 (Casp3). GzmB and caspase 3 target similar substrates that induce apoptosis of the infected cell. GzmA and GzmB enter the mitochondria – *via* Sam50 and Tim22 – where they target subunits of the electron transport chain (ETC) complex I, leading to the production of reactive oxygen species (ROS). The ROS favor the release of apoptogenic factors through the Bax/Bak pores, such as cytochrome C (Cyt C), Smac, Htr2A and endonuclease G (Endo G) in the cytosol. Cyt C binds Apaf1 to form the apoptosome, which matures the pro-caspase 9 (pro-Casp9) into active caspase 9 (Casp9). It is noteworthy that caspase 3, either activated by GzmB or caspase 9, also reaches the mitochondria – *via* Sam50 – where it cleaves a subunit of ETC complex I, leading to the production of ROS. Following these events, ROS concentration increases in the cytosol of the infected cell. The cytosolic ROS are involved in the translocation of the SET complex from the endoplasmic reticulum (ER) to the nucleus, where it is cleaved by GzmA and turns into a DNA degrading complex. GzmA and GzmB also reach the nucleus where they cleave nuclear substrates, such as lamin B, histones and PARP-1.

A key mechanism against intracellular pathogens, such as viruses, bacteria and parasites, is cell-mediated cytotoxicity exerted by killer lymphocytes of the innate and adaptive immune systems (1, 2). When these cytotoxic immune cells recognize cells infected with intracellular pathogens, they release their cytotoxic granule contents to eliminate the target cells and the intracellular pathogen. Cytotoxicity is mediated by a group of highly homologous serine proteases, the granzymes (Gzms), that are localized in specialized lysosomes of the killer cells (3). The best-established biological role of the Gzms is the induction of programed cell death when these cytotoxic proteases are delivered into the target cells by perforin (PFN) (4–6). PNF is a pore-forming protein (7) that facilitates the uptake of other cytolytic granule components by enhancing endocytic uptake (8–10) and promoting endosomolysis to allow cytosolic release (11) (**Figure 1**). Lymphocytic cytotoxic granules of humans and some other mammals, but not rodents, contain another membrane-disrupting protein, granulysin (GNLY) (14). GNLY belongs to the saposin-like protein family (SAPLIP) that is characterized by a particular polypeptide motif and its affinity to a variety of lipids (15). GNLY was found to disrupt prokaryotic (but not eukaryotic (16, 17)) membranes and to kill bacteria, parasites and fungi *in vitro* (18).

While the Gzms-mediated induction of host cell apoptosis is well established, the mechanisms of intracellular pathogen elimination is far less clear constituting an emerging field in recent years. The most obvious mechanism is that host cell death deprives obligate intracellular pathogens of their protective niche. Host cell death plays a major role in the elimination of many viruses (19) and obligate intracellular bacteria, such as *Chlamydia* spp (20, 21). These kind of pathogens counteract the host cell death machinery with a variety of inhibitory mechanisms, such as prevention of cytochrome C release (22) or the upregulation and/or mimicry of host anti-apoptotic proteins (23–25).

In addition to the induction of cell death, a direct mechanism of antimicrobial activities by the Gzms was discovered in numerous studies, which is mediated by the proteolytic degradation of microbial proteins to activate microbial death pathways, hence, limiting their growth inside a host, independently of host cell death. In this review, we aim to dissect these mechanisms with a particular focus on oxidative *versus* non-oxidative killing pathways.

## The Granzymes

The Gzms are a family of serine proteases firstly described by the team of Jürg Tschopp in 1986, who identified “granular enzymes” in the secretory granules of cytolytic lymphocytes (26). There are 5 human Gzms (A, B, H, K and M) and 10 mouse orthologues (A, B, C, D, E, F, G, K, M and N). The human Gzms are encoded from three different chromosomal loci: the chymase locus on chromosome 14 encodes for GzmB and GzmH, the met-ase locus on chromosome 19 for GzmM, and the tryptase locus on chromosome 5 harbors GzmA and GzmK (27). Although the human Gzms are highly homologous and share the catalytic triad (His^57^, Asp^102^ and Ser^195^), there are remarkable differences in their primary substrate specificities (28). The tryptases GzmA and GzmK cleave substrates after Arg or Lys (29, 30), GzmB cleaves after Asp (31), GzmH cleaves after Tyr or Phe (32), and GzmM cleaves after Leu or Met (33).

It is noteworthy that the Gzms, in particular GzmB, are not only expressed and secreted by killer lymphocytes. They were also detected in various non-cytotoxic immune cells (34), non-immune cells (35) and cancer cells (36–38). Interestingly, the non-cytolytic activities of granzyme B also modulate the differentiation of lymphoid cells *via* the interference with NOTCH1 signaling (39) or with production of IL-17 (40). As PFN is exclusively expressed in activated killer lymphocytes (41), the Gzms released from the above listed cells will exhibit predominantly extracellular effects. Potential activities include remodeling of extracellular matrix (42), modulating inflammation (43–45), detachment-mediated cell death, anoikis (46), and – as reviewed below – exerting antimicrobial activity against invading pathogens in synergy with antimicrobial peptides (AMPs) or by targeting secreted microbial proteins.

## The Granzymes in Cell Death

Due to this diversity in the cleavage site specificity, all Gzms have their unique degradomes (47), resulting in the activation of widely differing death pathways in target cells after cytosolic delivery by PFN. The best characterized death pathways are those of GzmA (48) and GzmB (49). GzmB executes death in a caspase-dependent manner (50) (**Figure 1**). Once released into the cytosol of a target cell, GzmB can directly cleave several caspases (51, 52), including caspase 3 (53). This executioner caspase trigger the release of an active DNase (CAD), responsible for DNA fragmentation upon various apoptotic stimuli (54). Human GzmB also efficiently cleaves the pro-apoptotic protein Bid (55). Truncated Bid induces Bad/Bax-dependent mitochondrial outer membrane permeabilization, the release of cytochrome C, SMAC/DIABLO, and other proteins, such as Htra2/omi, ultimately leading to apoptosome formation and activation of caspase 9 (56–61).

GzmA induces a cell death harboring morphological features similar to apoptosis: chromatin condensation, nuclear fragmentation, membrane blebbing, mitochondrial swelling and loss of cristae. However, GzmA does not activate executioner caspases to kill the cell. Cytosolic delivery of GzmA triggers a complex cascade of events that includes the translocation of a protein complex known as SET from the ER to the nucleus, ultimately leading to the nuclear transfer of two nucleases (NM23-H1 and Trex1) and lethal DNA damage (62–64). These pro-apoptotic features and mechanisms of GzmA were essentially established in the laboratory of Judy Lieberman (65).

An important common death mechanism of GzmA and GzmB is nuclear uptake to attack several nuclear proteins, involved in structural integrity, DNA repair and RNA splicing (66–72).

For the residual, “orphan” Gzms, caspase-dependency to induce death or even if the induction of apoptosis is their major function is still not clear and needs further study (73, 74). However, there are multiple lines of evidence suggesting that GzmM, GzmH and GzmK, as well as the non-orphan GzmA, have well defined proinflammatory and antimicrobial roles as further discussed below (75–78).

## Oxidative Cell Death Pathways Driven by the Granzymes

A critical common feature of GzmA and GzmB death pathways is the mitochondrial uptake of these enzymes (**Figure 1**). Once in the mitochondria, the Gzms cleave four subunits of the respiratory chain complex 1 (NDUFS3, NDUFV1, NDUFS1 and NDUFS2). The disruption of the electron transport chain dramatically increases premature electron leakage, leading to the formation of reactive oxygen species (ROS), a decrease in mitochondrial respiration and the loss of cristae (79–81). Strikingly, caspase 3 also degrades a complex 1 subunit (NDUFS1) to induce ROS-dependent cell death (82–84). In challenge of the orthodox mitochondrial import biology, GzmA and GzmB (and potentially caspase 3), without containing a canonical mitochondrial import sequence, cross the outer mitochondrial membrane through SAM50 channels and the inner membrane through TIM22 in a mtHSP70-dependent manner (85). The resulting increased ROS generation facilitates the release of apoptogenic factors through Bax/Bak pores and drives the nuclear translocation of the SET complex to enhance GzmB and GzmA death pathways, respectively. At least one study suggests that there might be additional, extra-mitochondrial sources of ROS induced by GzmB, in particular *via* the activation of NADPH-oxidase (86).

## Cytolytic Antimicrobial Functions of the Granzymes

The induction of host cell death *via* the granule exocytosis pathway is an obvious effector mechanism used by killer lymphocytes to eliminate the host cells and obligate intracellular pathogens, such as viruses (87) and certain unicellular parasites (88) or bacteria (89). Suicidal death is an approved defense mechanism of cells infected with pathogens independently of a lymphocyte attack (90–92). Programed host cell death deprives the pathogens of their protective niche, minimizes the risk of dissemination as membrane integrity is initially preserved, and recruits and activates phagocytes to digest the remains (93). Therefore, it is not surprising that obligate intracellular pathogens evolved multiple mechanisms to counteract the death machinery, as already documented in a vast body of comprehensive reviewing literature (87, 94–97). More interesting in this particular context, the Gzms are capable to digest vital microbial substrates independently of host cell death that can directly affect pathogen survival as discussed below in the main focus of this reviewing article.

## Non-Cytolytic Antimicrobial Functions of the Granzymes

Non-cytolytic, direct antimicrobial activities by the Gzms were primarily demonstrated in virus-infected cells (2). When the Gzms enter a virus-infected cell in a PFN-dependent manner, they will induce apoptosis to deprive the virus of its protective niche as described above. The induction of programed cell death is often inefficient as many viruses evolved multiple pathways to inhibit the death machinery by means of caspase or Gzms inhibition, as well as by mimicking anti-apoptotic proteins, such as Bcl-2 (98–101). Nevertheless, the Gzms can effectively overcome this inhibition by targeting viral proteins or host proteins hijacked by the virus involved in viral replication. The laboratory of Markus Simon previously demonstrated that mouse GzmA cleaved and therefore inactivated the enzymatic activity of reverse transcriptase from Moloney murine leukemia virus. As reverse transcriptase activity is critical for the retroviral life cycle, GzmA might potentially interfere with retroviral replication (102).

In a report concerning adenovirus, GzmH was shown to proteolytically degrade adenovirus DNA-binding protein (DBP), a crucial viral component DNA replication (103). Interestingly, GzmH additionally directly inactivated L4-100K assembly protein crucial for viral assembly and also a potent inhibitor of GzmB (104), suggesting complex interaction of these serine proteases in virus-infected cells.

Also for the family of *Herpesviridae*, such as human cytomegalovirus (HCMV) or herpes simplex virus-1 (HSV-1), multiple viral substrates were identified that are targeted by the Gzms. GzmM interferes with HCMV replication independently of cell death by the proteolytic degradation of phosphoprotein 71, a HCMV protein critical for immediate-early protein expression (105). In more recent works, the same laboratory demonstrated that virus-specific T cells control HCMV replication in a non-cytolytic manner by the Gzms-mediated degradation of the HCMV immediate early proteins IE1 and IE2 (106), as well as of host cell hnRNP K, essential for HCMV replication (107).

GzmA deficiency in mice was associated with impaired control of HSV-1 in infected neurons (108). In addition, human GzmB cleaves the HSV-1 immediate-early protein ICP4, therefore potentially contributing to the non-cytolytic inhibition of viral reactivation in latently infected ganglion cells, mediated by HSV-1 specific T cells (109). In a more recent study, several novel HSV-1 GzmB substrates were identified, suggesting an even broader non-cytolytic role of GzmB in the control of *Herpesviridae* (110).

In conclusion, non-cytolytic, direct antimicrobial activities of the Gzms against viruses are well established. Viral substrates or, for viral replication, essential host factors that are targeted by the Gzms were identified for many additional viruses, such as vaccinia (111), hepatitis C (112) and hepatitis B (113), as well as influenza A virus (114).

**Figure 2:**
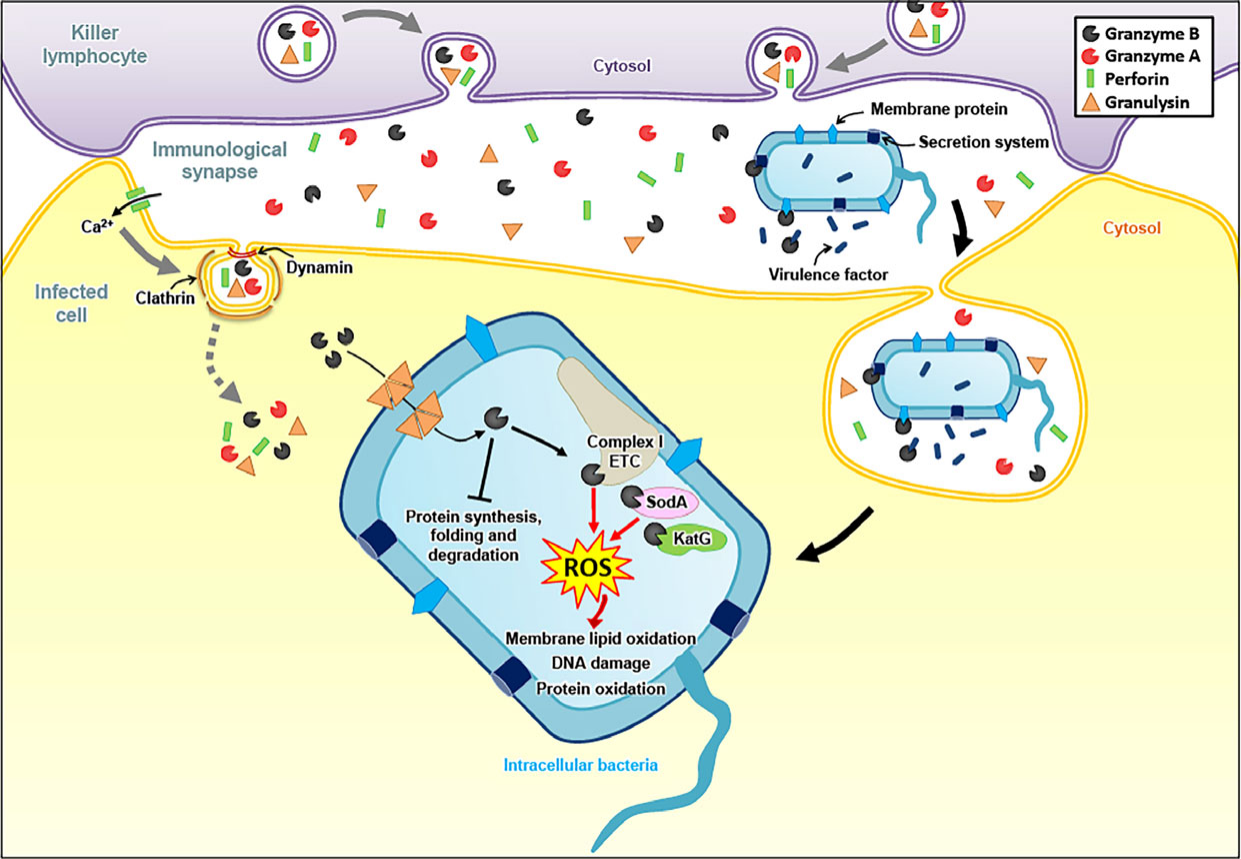
Granzyme-mediated death pathways in bacteria. To fight intracellular bacteria, Gzms and GNLY are delivered into infected cells in a PFN dependent manner. GNLY then forms pores in bacterial membranes, allowing the entry of the Gzms into the bacterial cytosol. GzmB cleaves the catalytic subunits of electron transport chain (ETC) complex I, as well as bacterial proteins involved in antioxidant defense, generating ROS that induce membrane lipid oxidation, DNA damage and protein oxidation. GzmB also target various bacterial proteins involved in protein synthesis, folding and degradation. Independently of GNLY, extracellular GzmB directly targets external bacterial components, such as secretion systems, membrane proteins and secreted virulence factors to attenuate virulence and, consequently, to facilitate bacterial elimination in bystander cells.

Less is known for other intracellular pathogens, such as bacteria or parasites. In earlier work, we found that activating human naïve T cells with bacterial antigens not only triggered the expression of known antibacterial effectors, such as GNLY, interferon-γ or tumor necrosis factor-α, but also of the Gzms in remarkable high levels, in particular GzmB (115). These findings corroborated a previous study indicating elevated plasma levels of GzmA and GzmB in patients with bacterial infections or after endotoxin administration (116). By following up these works, we realized through functional antibacterial assays that GzmA, GzmB and GzmM (the other human Gzms were not tested) are potent antibacterial effectors when these enzymes are delivered into bacteria by GNLY (117, 118) (**Figure 2**). Building on these findings, a recent study demonstrated that the high levels of secreted GzmB and GNLY by activated mucosa-associated invariant T cells (MAIT) not only directly damage bacteria but also increase the susceptibility to carbapenems in multidrug resistant *E. coli* (119, 120). In addition, it was revealed that the three major effector molecules of cytotoxic lymphocytes (Gzms, GNLY and PFN) collaborate in a highly coordinated manner to kill intracellular bacteria, such as *Listeria monocytogenes* (121). The proteolytic activity of the Gzms is necessary to achieve this function, as mutations of their catalytic site impaired the killing of intracellular pathogens. These mutations were introduced by using a mammalian expression system, allowing the generation of comparable purifications of catalytically active and non-active Gzms (122). An unbiased proteomics search for GzmB substrates in several bacterial strains revealed a well-defined list of bacterial proteins, involved in multiple critical metabolic pathways, including protein synthesis and virulence (123). Indeed, extracellular Gzms degraded secreted bacterial virulence factors in absence of GNLY, overall decreasing virulence of the affected bacteria, therefore enabling bystander immune and non-immune cells to more efficiently eliminate the invading pathogens (124).

**Figure 3:**
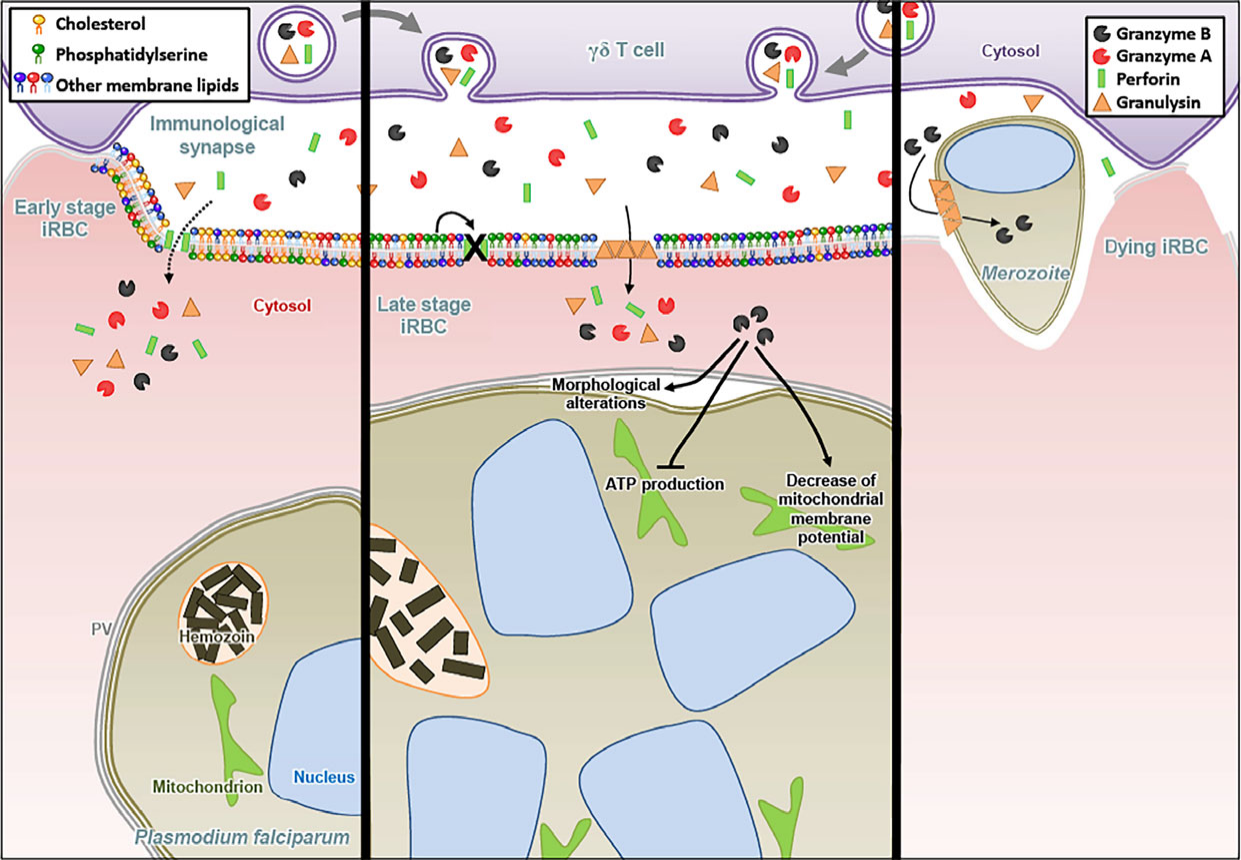
Granzyme B released by γδ T cells contributes to anti-malaria defense. The particular killer lymphocyte subset bearing the γδ T receptor forms an immunological synapse with *Plasmodium falciparum*-infected red blood cells (iRBC). In early stage iRBC, the plasma membrane contains cholesterol-enriched lipid rafts and the negatively charged phosphatidylserine (PS) is predominantly present in the inner leaflet. At that early stage, PFN can form membrane pores allowing the entry of Gzms, while being resistant to GNLY lysis. For late stage iRBC, cholesterol depletion allows the GNLY to disrupt the membrane while the surface exposure of PS inhibits the formation of PFN pores. Once in the iRBC, GzmB induces dramatic morphological alterations of late stage parasites (schizonts), notably the detachment of the parasitophorous vacuole (PV). Moreover, GzmB inhibits ATP production and decreases the mitochondrial membrane potential of the parasite. At the end of the parasite growth cycle, the rupture of iRBC plasma membrane leads to merozoites egress. GNLY also disrupts the membrane of the merozoite, allowing the entry of GzmB in the parasite.

**Figure 4:**
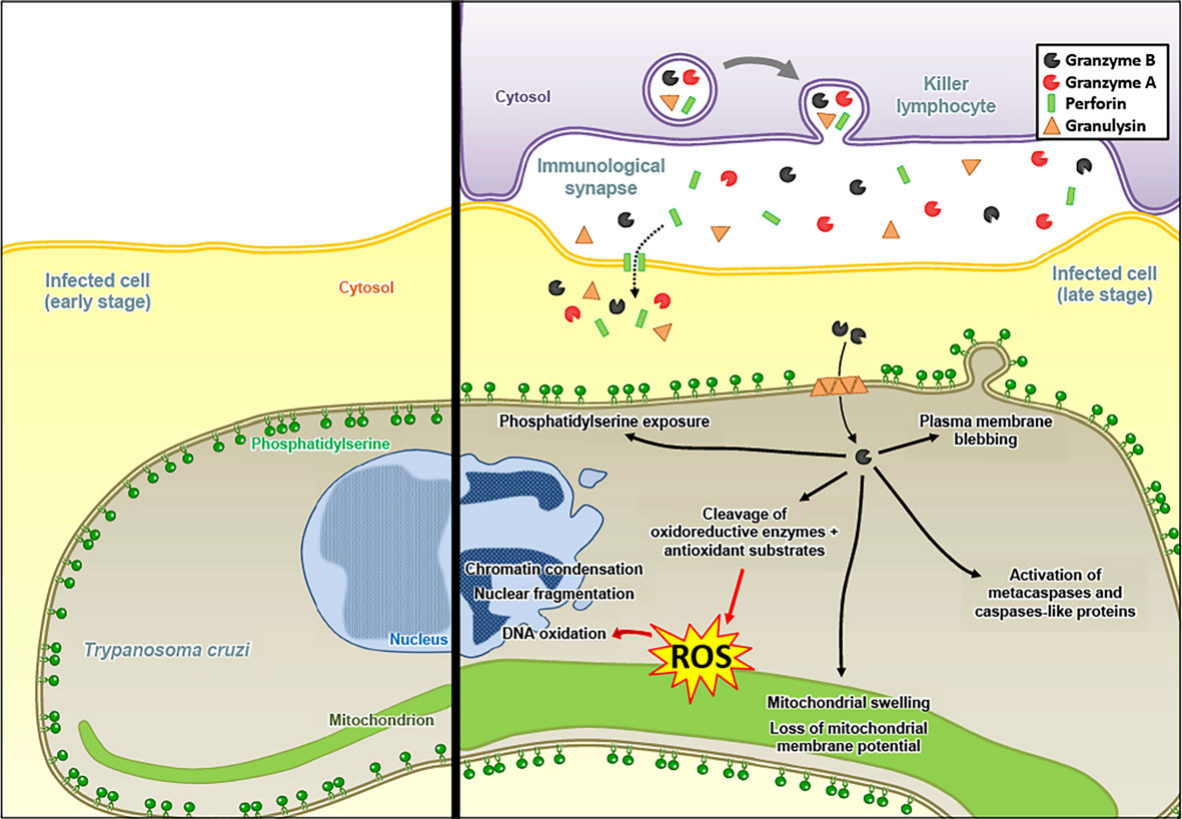
Granzyme B induces an apoptosis-like death pathway in *Trypanosoma cruzi*. The Gzms of killer lymphocyte granules reach the cytosol of *T. cruzi* in an infected host cell *via* a PFN- and GNLY-dependent delivery mechanism. After entry into the parasites, GzmB targets antioxidant defense substrates, which leads to the accumulation of ROS, responsible for DNA oxidation. GzmB triggers classical mammalian apoptosis features: (1) chromatin condensation and nuclear fragmentation, (2) mitochondrial swelling and loss of membrane potential, (3) plasma membrane blebbing, and (4) phosphatidylserine exposure. GzmB also induces the activation of metacaspases and caspase-like proteins, although it might not be required for GzmB-mediated parasite death.

Interestingly, Gzms-mediated killing mechanisms after delivery by PFN and GNLY were also found against certain unicellular parasites, such as *Plasmodium falciparum* (125, 126). For the *Plasmodium* parasite, we found that the mechanism of Gzms delivery changed upon maturation of the intracellular pathogen in red blood cells (RBCs). While early stage infected RBCs (rings and trophozoites) are susceptible to PFN and resistant to GNLY, late stages (schizonts) display the opposite behavior due to membrane cholesterol depletion and increased phosphatidylserine exposure upon parasite maturation (**Figure 3**) (127). Also for *Toxoplasma gondii* and *Trypanosoma cruzi*, a PFN- and GNLY-dependent delivery mechanism of the Gzms was revealed that induced a death pathway in the parasites, displaying morphological features highly similar to mammalian apoptosis (**Figure 4**) (128).

## Oxidative Antimicrobial Functions of the Granzymes

As for the cytolytic activities of the Gzms, the induction of ROS seems to be a critical merging point in the antimicrobial mechanisms against various pathogens. In *E. coli*, the Gzms attacked homologue subunits of the respiratory chain complex-1, as in mammalian mitochondria, suggesting an evolutionary well-conserved killing mechanism. The premature electron leakage from the disrupted respiratory machinery in combination with the GzmB proteolysis of important antioxidant defense enzymes, such as superoxide dismutase and catalase, triggered lethal ROS levels in the affected bacteria (118). GzmB also extensively targeted the ROS defense machinery in the proteome of *Mycobacteria tuberculosis*, *Listeria monocytogenes*, as well as *Salmonella typhimurium*, suggesting that oxidative mechanisms also play a central role in the GzmB-mediated death pathways in these bacterial pathogens (123, 124). Major antioxidant enzymes were also degraded by GzmB in the unicellular parasites, *Plasmodium falciparum, Toxoplasma gondii* and *Trypanosoma cruzi* (125, 128). For the latter parasites, a dominant role of ROS in the killing mechanism was indicated by several lines of evidence: 1. ROS were produced in response to GzmB after delivery with pore forming proteins, 2. ROS scavengers efficiently inhibited the killing, and 3. GzmB uncleavable point mutations in major antioxidant defense enzymes slowed down the death pathway (128). GzmB delivery into unicellular parasites also clearly affect the mitochondria as indicated in morphological alterations, loss of mitochondrial membrane potential and decreased ATP production (125, 128).

## Conclusions

Though this particular field of research is only developing and further study is necessary, we think it is fair to state that the Gzms exert potent antimicrobial activities by direct proteolysis of vital microbial substrates that are crucial for their replication. Best studied so far in virus-infected cells; however, numerous studies indicate that the Gzms can also lethally affect intracellular bacteria and unicellular parasites by means that are independent of host cell cytolysis. Mitochondria and increased ROS generation seem to be on center stage in Gzms-mediated death pathways in mammalian cells and unicellular parasites. As mitochondria originated from endosymbiotic alpha-proteobacteria, it was not surprising to find respiratory chain disruption by the Gzms in modern living bacteria. To what exact extent these ROS contribute to the killing pathways of the different microbes and mammalian cells is still a matter of debate and needs further study. Nonetheless, there is little doubt that ROS pathways seem to be a highly conserved target in Gzms-mediated death pathways in various species that are evolutionarily far apart.

## Author Contributions

MW conceived the concept. ML, MH-C, P-YM, DM and MW researched the literature, wrote, edited and revised the manuscript. ML designed and illustrated the figures. All authors contributed to the article and approved the submitted version.

## Funding

This work was supported by the Swiss National Science Foundation (SNSF grant # 310030_169928), the Novartis Foundation for Medical-Biological Research (Novartis grant # 20B136), and the Vontobel-Foundation (all to MW). The funders were not involved in the study design, collection, analysis, interpretation of data, the writing of this article or the decision to submit it for publication.

## Conflict of Interest

The authors declare that the research was conducted in the absence of any commercial or financial relationships that could be construed as a potential conflict of interest.

## Publisher’s Note

All claims expressed in this article are solely those of the authors and do not necessarily represent those of their affiliated organizations, or those of the publisher, the editors and the reviewers. Any product that may be evaluated in this article, or claim that may be made by its manufacturer, is not guaranteed or endorsed by the publisher.
